# Aphasia

**Published:** 1907-03-23

**Authors:** 


### Aphasia.

In a science like pathology that is continually progressing, we must always be prepared to re-examine accepted theories in the light of fresh researches, and, if necessary, to modify or even remodel them anew. One of the best-established facts in neuropathology has been the localisation of the functions of speech in the cerebral hemispheres. Broca first laid the foundation-stone when in 1861 he showed the association of motor aphasia with a lesion of the posterior portion of the third frontal convolution of the left cerebral hemisphere, now known as Broca's area. Later (in 1874) Wernicke demonstrated a sensory centre for language situated in the supra-marginal and angular convolutions, destruction of which was followed by a failure to comprehend language, spoken or written. By the researches of Charcot, Exner, Déjerine, Ferrier, and others, further distinctions in aphasic conditions were established, so that we now have alexia, agraphia or word blindness, word deafness, and the subcortical aphasias due to lesions of the white matter underlying the cortex and involving chiefly the association fibres between the different centres.

Professor Pierre Marie, of the great hospital at Bicêtre, who enjoys a high reputation as a neurologist, now seeks completely to overthrow these theories in regard to the localisation of the centres for the functions of speech. By an extended series of researches, in which he claims to have examined nearly one hundred cases of aphasia, and to have made more than fifty autopsies, he is led to conclude that Broca's convolution is in no way connected with the function of speech. In criticising the original cases, upon which Broca founded his theory, he contends that in one case the lesion extended over a wide area, involving not only the third frontal convolution, but also a large portion of the parietal and temporal convolutions, and including parts of the supra-marginal and of the angular gyri. In another case the third frontal convolution was not involved at all, but Broca failed to recognise a condition of senile atrophy of the brain. Marie associates aphasia entirely with the area of Wernicke—that is the gyrus supra-marginalis, the angular gyrus, and the posterior parts of the first and second temporal convolutions. He explains the frequent occurrence of a lesion of Broca's area with the phenomena of aphasia, on the ground that obliteration of the middle cerebral artery generally takes place above the origin of the branch to this area, so that this convolution is involved together with those parts, mentioned above, in which the function of speech is actually situated. He affirms that there are cases in which the destruction of Broca's area has not been followed by aphasia, and on the other hand asserts that he has records of cases of typical Broca's aphasia, in which Broca's area was intact.

Marie denies that there is any evidence in favour of a centre of hearing in the first temporo-sphenoidal convolution; and, in fact, puts his pen through all the nice distinctions that have been made between the various forms of sensory and motor aphasia, corresponding to the various centres or association fibres involved in the different lesions. He recognises the aphasia of Wernicke, in which there is more or less loss of comprehension of speech and of general intelligence, varying according to the extent of the area involved in the lesion. From this aphasia he distinguishes what he terms anarthria, or pure motor aphasia. Those afflicted with anarthria can read, write, and understand what is said or written, but they are unable to articulate words, although they can indicate by signs the letters or syllables of which the words are composed. This anarthria of Marie corresponds to the subcortical motor aphasia of Déjerine. The lesion of anarthria is localised in the region of the lenticular nucleus or in the anterior part of the knee of the internal capsule.

According to Marie, Broca's motor aphasia is simply Wernicke's aphasia, or the inability to comprehend language plus anarthria, so that we should only have three distinguishing forms of defective function of speech—aphasia (Wernicke's sensory aphasia), aphasia and anarthria (Broca's motor aphasia), and anarthria.

Marie's theories will undoubtedly be subjected to much searching criticism before they are either finally adopted or rejected. Cases of aphasia will receive the closest study both clinically and pathologically. Already, since the publication of his article, two cases have been placed on record, which appear to substantiate his views. Whatever may be the ultimate issue in regard to the facts, it is to be regretted that he should have adopted the term anarthria for a form of pure motor aphasia, seeing that this word has already acquired a different significance. Anarthria has hitherto been used to signify an inability to articulate owing to a paralysis of the muscles of articulation, such as occurs in conditions of bulbar paralysis. In the condition designated anarthria by Marie the muscular apparatus is intact, but the individual has lost the power to employ it to express his ideas. Such a misuse of terms is only likely to lead to needless discussions and misunderstandings.

